# Supplementary material for: Resolving the full spectrum of human genome variation using Linked-Reads
Source: Genome Res. 2019 Apr;29(4):635–45. doi: 10.1101/gr.234443.118 (PMC6442396; doi:10.1101/gr.234443.118)
Supplement: Supplemental Material [file supp_gr.234443.118_Supplemental_longranger-2.2.zip › longranger-2.2.tar.bz2!/lr2.2-public/lib/go/src/code.google.com/vitess/docs/user-guide/row-based-replication.html]

Vitess / Row Based Replication


Toggle navigation

- Overview
- Guides
- Reference
- Blog
- GitHub

- #### Overview

  - What is Vitess
  - Scaling MySQL with Vitess
  - Key Concepts
- #### Getting Started

  - Run Vitess on Kubernetes
    - Custom Docker Build
  - Run Vitess Locally
- #### User Guide

  - Introduction- Client Libraries- Backing Up Data- Reparenting
        - Schema Management
        - VSchema Guide
        - Schema Swap (Tutorial)
        - Sharding
          - Horizontal Sharding (Tutorial, automated)
          - Horizontal Sharding (Tutorial, manual)
          - Sharding in Kubernetes (Tutorial, automated)
          - Sharding in Kubernetes (Tutorial, manual)
        - Vitess Sequences
        - MySQL Server Protocol
        - Vitess and Replication
        - Update Stream
        - Row Based Replication
        - Topology Service
        - Transport Security Model
        - Launching
          - Scalability Philosophy
          - Production Planning
          - Server Configuration
          - 2PC Guide
          - Troubleshooting
        - Upgrading
- #### Reference Guides

  - Vitess API- vtctl Commands
- #### Other Resources

  - Presentations- Blog- Roadmap
- #### Contributing

  - Contributing to Vitess- GitHub Workflow- Code Reviews
- GitHub

# Row Based Replication

- #### Overview

  - What is Vitess
  - Scaling MySQL with Vitess
  - Key Concepts
- #### Getting Started

  - Run Vitess on Kubernetes
    - Custom Docker Build
  - Run Vitess Locally
- #### User Guide

  - Introduction- Client Libraries- Backing Up Data- Reparenting
        - Schema Management
        - VSchema Guide
        - Schema Swap (Tutorial)
        - Sharding
          - Horizontal Sharding (Tutorial, automated)
          - Horizontal Sharding (Tutorial, manual)
          - Sharding in Kubernetes (Tutorial, automated)
          - Sharding in Kubernetes (Tutorial, manual)
        - Vitess Sequences
        - MySQL Server Protocol
        - Vitess and Replication
        - Update Stream
        - Row Based Replication
        - Topology Service
        - Transport Security Model
        - Launching
          - Scalability Philosophy
          - Production Planning
          - Server Configuration
          - 2PC Guide
          - Troubleshooting
        - Upgrading
- #### Reference Guides

  - Vitess API- vtctl Commands
- #### Other Resources

  - Presentations- Blog- Roadmap
- #### Contributing

  - Contributing to Vitess- GitHub Workflow- Code Reviews

# Row Based Replication

In Vitess 2.2, we are adding preliminary support for Row Based Replication. This
document explains how we are managing it and how it affects various Vitess
features.

See the Vites and Replication document
for an introduction on various types of replication and how it affects Vitess.

## MySQL Row Based Replication

With Row Based replication, a more compact binary version of the rows affected
are sent through the replication stream, instead of the SQL statements. The
slaves then do not spend any time parsing the SQL, or performing any complex SQL
operations (like `where` clauses). They can just apply the new rows directly.

A few binlog events are used:

- Table Map event: describes a table that is affected by the next
  events. Contains the database and table name, the number of columns, and the
  Type for each column. It does not contain the individual column names, nor the
  flags for each column (so it is impossible to differentiate signed vs unsigned
  integers for instance).
- Write Rows: equivalent of Insert.
- Update Rows: change the values of some rows.
- Delete Rows: delete the provided rows.

The
`binlog-row-image` option can
be used to control which rows are used to identify the columns for the Update
and Delete Rows events. The default setting for that option is to log all
columns.

## Vitess Use of MySQL Replication Stream

Vitess uses the Replication Stream in a number of places. This part explains how
we use RBR for these.

### vttablet Replication Stream Watcher

This is enabled by the `watch_replication_stream` option, and is used
by Update Stream. It only cares about the
GTIDs for the events, so it is unaffected by the use of RBR.

*Note*: the current vttablet also reloads the schema when it sees a DDL in the
stream. See below for more information on this. DDLs are however not represented
in RBR, so this is an orthogonal issue.

### Update Stream

The current implementation uses comments in the original SQL (in SQR) to provide
the primary key of the column that is being changed.

We are changing this to also parse the RBR events, and extract the primary key
value.

*Note*: this means we need accurate schema information. See below.

### Filtered Replication

This is used during horizontal and vertical resharding, to keep source and
destination shards up to date.

We need to transform the RBR events into SQL statements, filter them based
either on keyspace\_id (horizontal resharding) or table name (vertical
resharding), and apply them.

For horizontal splits, we need to understand the VSchema to be able to find the
primary VIndex used for sharding.

*Note*: this again means we need accurate schema information. We could do one of
two things:

- Send all statements to all destination shards, and let them do the
  filtering. They can have accurate schema information if they receive and apply
  all schema changes through Filtered Replication.
- Have the filtering be done on the stream server side, and assume the schema
  doesn't change in incompatible ways. As this is simpler for now, that's the
  option we're going with.

## Database Schema Considerations

### Interpreting RBR Events

A lot of the work to interpret RBR events correctly requires knowledge of the
table's schema. However, this introduces the possibility of inconsistencies
during schema changes: the current schema for a table might be newer than the
schema an older replication stream event was using.

For the short term, Vitess will not deal very gracefully with this scenario: we
will only support the case where the current schema for a table has exactly the
same columns as all events in the binlog, plus some other optional columns that
are then unused. That way, it is possible to add columns to tables without
breaking anything.

Note if the main use case is Filtered Replicaiton for resharding, this
limitation only exists while the resharding process is running. It is somewhat
easy to not change the schema at the same time as resharding is on-going.

### Applying Schema Changes

When using
RBR, Schema Swap
becomes useless, as replication between hosts with different schemas will most
likely break. This is however an existing limitation that is already known and
handled by MySQL DBAs.

Vitess at this point does not provide an integrated way of applying involved
schema changes through RBR. A number of external tools however already exist to
handle this case, like gh-ost.

We have future plans to:

- Integrate with a tool like gh-ost to provide a seamless schema change story.
- Maintain a history of the schema changes that happen on all shards, so events
  can be parsed correctly in all cases.

## Unsupported Features

This part describes the features that are not supported for RBR in Vitess as of
March 2017:

- *Fractional timestamps for MariaDB*: not supported. This affects the objects
  of type `TIMESTAMP`, `TIME` and `DATETIME`. The way that feature is
  implemented in MariaDB, the binary logs do not contain enough information to
  be parsed, but instead MariaDB relies on the schema knowledge. This is very
  fragile. MySQL 5.6+ added new data types, and these are supported.
- *JSON type in MySQL 5.7+*: the representation of these in the binlogs is a
  blob containing indexed binary data. Re-building the SQL version of the data,
  so it can be re-inserted during resharding, is not supported yet. It wouldn't
  however be a lot of work, with other libraries also supporting this, and the
  C++ MySQL code being well written and easy to read. See for instance
  https://github.com/shyiko/mysql-binlog-connector-java/pull/119
- *Timezones support*: the binary logs store timestamps in UTC. When converting
  these to SQL, we print the UTC value. If the server is not in UTC, that will
  result in data corruption. *Note*: we are working on a fix for that one.

## Update Stream Extensions

Update Stream can be changed to contain both
old and new values of the rows being changed. Again the values will depend on
the schema. We will also make this feature optional, so if the client is using
this for Primary Key based cache invalidation for instance, no extra unneeded
data is sent.

This can be used to re-populate a cache with Update Stream, instead of
invalidating it, by putting the new values directly in there.

Then, using this in conjunction with `binlog-row-image` would help provide a
feature-complete way of always getting all changes on rows. It would also help
handle Update Stream corner cases that replay events during resharding, when
switching traffic from old to new shards.

## Vttablet Simplifications

A lot of the work done by vttablet now is to find the Primary Key of the
modified rows, to rewrite the queries in an efficient way and tag each statement
with the Primary Key. None of this may be necessary with RBR.

We plan to eventually add a `rbr_mode` flag to vttablet to disable all the
things it can skip if RBR is used.

Contact: vitess@googlegroups.com  **·**  
Announcements  **·**  
© 2017 Vitess powered by Google Inc
